# Patient-operated imaging for clinical assessment: regulatory and reimbursement challenges in European health systems

**DOI:** 10.3389/fdgth.2026.1854287

**Published:** 2026-07-01

**Authors:** Chifra Fenton, Charles Tibi, Jacob Glazer, Nadia Prisant

**Affiliations:** 1 Department of Radiology, Tel Aviv Sourasky Medical Center, Tel Aviv, Israel; 2 Faculty of Medicine, Tel Aviv University, Tel Aviv, Israel; 3 Department of Obstetrics and Gynecology, Reproductive Medicine, American Hospital of Paris, Neuilly, France; 4 Department of Economics, University of Warwick, Coventry, United Kingdom; 5 Coller School of Management, Tel Aviv University, Tel Aviv, Israel; 6 Department of Pathology, Sheba Medical Center, Tel Hashomer, Ramat Gan, Israel; 7 Medical Affairs Department, IMMA Health, Modiin, Israel

**Keywords:** fertility monitoring, health technology assessment (HTA), medical device regulation (MDR), patient-operated imaging, point of care ultrasound (POCUS), reimbursement policy, telemedicine

## Abstract

**Background:**

Patient-operated medical technologies are increasingly integrated into European care. Under EU regulation, CE marking confirms safety and performance for each specific intended use, while reimbursement requires separate demonstration of clinical and economic value. Imaging has historically differed because acquisition and interpretation are typically coupled within the same clinical encounter.

**Objective:**

To examine patient-operated imaging for clinical assessment (POICA) as a regulatory and reimbursement boundary case under the EU Medical Device Regulation (MDR), and to analyse how redistribution of image acquisition to patients, while preserving clinician-led interpretation, challenges regulatory classification, liability allocation, and reimbursement models historically built around a unified professional imaging act.

**Methods:**

We conducted a structured policy and regulatory analysis examining how patient-operated imaging interacts with existing European regulatory and reimbursement frameworks. Sources were selected based on their relevance to decentralized acquisition, clinical reliability, workflow organization, and reimbursement integration. The analysis integrates MDR and Medical Device Coordination Group (MDCG) guidance documents, usability and risk-management standards), liability principles for home-use medical devices, and the structure of European health technology assessment (HTA) and reimbursement systems. Patient-operated imaging was evaluated against precedents in which elements of technical execution moved outside institutional settings (e.g., self-testing *in vitro* diagnostics, home-based therapies, and remote monitoring models) to identify where existing frameworks accommodate decentralized image acquisition and where structural tensions arise.

**Results:**

Imaging, even in structured monitoring contexts such as fertility care, requires each acquisition to meet clinical adequacy thresholds at the time of interpretation. This creates acquisition-dependent risk that cannot be mitigated through repetition alone. Under the MDR, adequacy criteria and the mechanisms ensuring them must be explicit; usability validation must demonstrate interpretative reliability rather than procedural completion. Responsibility becomes hybrid, with patients performing acquisition while clinicians retain interpretive and clinical decision-making authority. Economically, this redistribution disrupts reimbursement models built around a single bundled professional imaging act.

**Conclusions:**

The central challenge is not regulatory authorization but maintaining clinical reliability when acquisition is decentralized. Integration into European health systems will depend on demonstrable reliability of patient-acquired images, clear allocation of responsibility, and reimbursement mechanisms capable of recognizing distributed clinical workflows, while maintaining alignment between physician incentives and high-quality care.

## 1 Introduction

Patient-operated technologies have moved parts of data generation outside hospitals. In Europe, CE marking under the Medical Device Regulation (MDR and MDCG guidance documents) confirms that a device meets defined safety and performance requirements for its intended use (1–3). Reimbursement, however, follows a separate logic: health systems require demonstration of clinical utility and economic value before coverage decisions are made.

Imaging has historically followed a different model. In this traditional model, acquisition and interpretation are performed as a single clinical act within the same encounter. Radiological policy frameworks describe imaging as a specialist medical activity performed within institutional governance and professional training structures (4). This organizational coupling has shaped both regulatory expectations and reimbursement models.

Over the past decade, imaging has begun to decentralize. The expansion of point-of-care ultrasound (POCUS) shows that image acquisition can move beyond radiology departments to clinicians in emergency medicine, obstetrics, cardiology, and primary care while interpretation remains medical (5–7).

The recent development of handheld ultrasound systems has further accelerated this decentralization of imaging capabilities (8). Advances in handheld ultrasound devices, automated acquisition guidance, and tele-ultrasound platforms have expanded the possibility of performing ultrasound examinations outside traditional clinical environments (9).

These developments illustrate a shift in clinical workflows in which elements of data acquisition increasingly occur outside hospitals while clinical interpretation remains under professional supervision (10).

The increasing use of teleradiology has already introduced a degree of separation between image acquisition and interpretation in routine practice, with radiologists frequently interpreting images acquired at distant sites (11). Patient-operated imaging for clinical assessment (POICA) represents a further step in this evolution, extending this separation beyond healthcare professionals to patients (Table 1). In this configuration, clinicians retain interpretive authority while the technical act of acquisition is performed by the patient. This redistribution raises new questions for regulatory classification, usability validation, liability allocation, and reimbursement models historically organized around a single clinical imaging act.

**Table 1:** Comparative characteristics of imaging acquisition models.

Model	Image acquisition	Image interpretation	Organizational configuration	Clinical responsibility
Conventional imaging	Radiology staff/clinician	Radiologist or clinician	Co-localized acquisition and interpretation	Integrated
Point-of-care ultrasound (POCUS)	Treating clinician	Same clinician	Decentralized clinician-operated imaging	Integrated
Teleradiology	Local healthcare professional	Remote radiologist	Geographically separated interpretation	Shared workflow
Patient-operated imaging (POICA)	Patient	Physician	Decentralized patient acquisition	Hybrid

**Figure 1:**
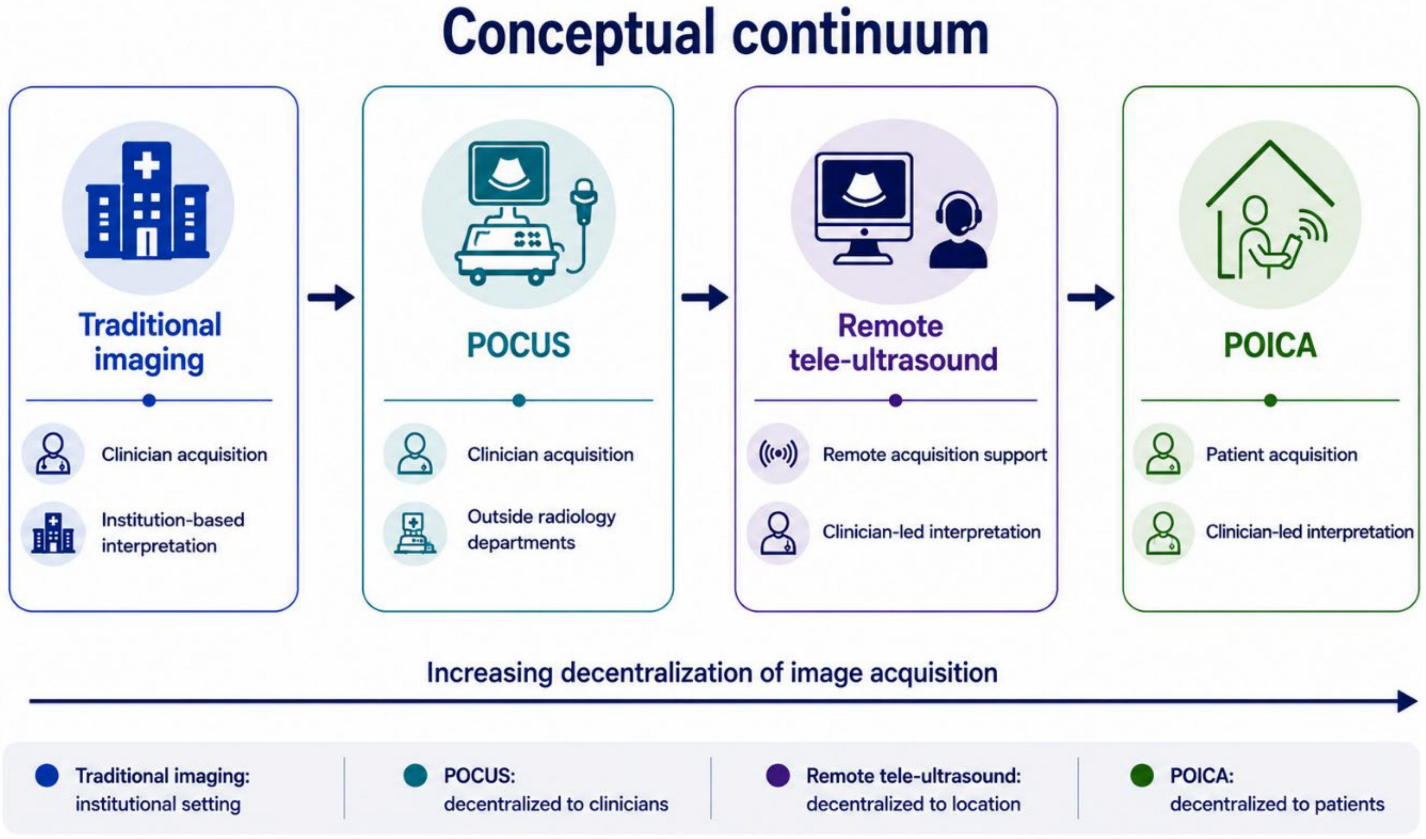
Conceptual continuum of imaging acquisition models. Imaging acquisition models have progressively redistributed the technical act of acquisition beyond conventional institutional environments. Traditional imaging bundles acquisition and interpretation within hospital-based clinical workflows. Point-of-care ultrasound (POCUS) extended acquisition to clinicians outside radiology departments while maintaining clinician-led interpretation. Remote tele-ultrasound further enabled acquisition outside hospitals through remote or guided workflows. Patient-operated imaging for clinical assessment (POICA) represents a further decentralization step in which image acquisition is performed by patients while interpretation and clinical decision-making remain clinician-led.

The progressive redistribution of imaging acquisition can be conceptualized as a continuum from institutional imaging to clinician-operated point-of-care imaging and finally to patient-operated acquisition (Figure 1).

These models are not only conceptual. In clinical practice, POCUS illustrates clinician-operated decentralized acquisition, where the treating clinician performs and interprets ultrasound at the bedside within a defined clinical context (5). Teleradiology illustrates geographical separation between acquisition and interpretation, with images acquired locally and interpreted remotely by radiologists under established quality and responsibility frameworks (11). Patient-operated ultrasound has also been evaluated in structured reproductive care, including self-operated endovaginal telemonitoring during ovarian stimulation monitoring (12). These examples suggest that the relevant policy question is not whether acquisition and interpretation can ever be separated, but how far redistribution of acquisition can extend while maintaining acquisition adequacy, clinical reliability, accountability, and reimbursement coherence.

We examine these questions through the lens of **fertility monitoring**, a bounded and protocol-driven clinical context in which patient-operated acquisition can be evaluated without extending the discussion to unrestricted diagnostic imaging.

The central policy question raised by patient-operated imaging is therefore not primarily technological. Ultrasound devices capable of guided acquisition already exist, and decentralized monitoring models are emerging in several clinical fields. The policy challenge lies in how existing regulatory and reimbursement frameworks accommodate a model in which the technical act of image acquisition is performed outside the clinical encounter while interpretation and clinical responsibility remain physician-led. This redistribution of the imaging act sits at the boundary of current regulatory classifications and reimbursement structures.

However, before examining regulatory and reimbursement implications, we must first define what POICA represents within existing medical device and care delivery frameworks.

### Policy analysis approach

This manuscript presents a structured policy and regulatory analysis rather than a systematic review or formal health technology assessment. Sources were identified through targeted review of European regulatory guidance documents (MDR and MDCG guidance), usability and risk-management standards, and literature related to decentralized care models, teleradiology, remote monitoring, self-testing diagnostics, and reimbursement structures. Sources were selected based on their relevance to redistribution of technical acquisition outside institutional settings and their implications for clinical reliability, responsibility allocation, workflow organization, and reimbursement integration. Comparative analogies from existing decentralized healthcare models were used to identify where current frameworks accommodate patient-operated acquisition and where structural tensions remain.

## 2 Conceptual framework: patient-operated imaging for clinical assessment (POICA)

Patient-operated imaging for clinical assessment (POICA) refers to an organizational model in which image acquisition is performed by the patient, often outside a conventional clinical setting, while image interpretation and clinical decision-making remain clinician-led. It does not refer to autonomous diagnosis (13).

POICA is distinct from point-of-care ultrasound (POCUS), tele-ultrasound, teleradiology, and conventional home monitoring models. In POCUS, acquisition and interpretation are performed by a clinician at the bedside. In tele-ultrasound and teleradiology, acquisition remains performed by trained healthcare professionals even when interpretation occurs remotely. In POICA, by contrast, the technical acquisition act itself is redistributed to the patient while interpretation and clinical responsibility remain physician-led.

The defining feature is not who makes clinical decisions, but who performs the technical act of acquisition. The distinction is organizational rather than diagnostic: professional responsibility is preserved, but the location and executor of the technical act change.

POICA sits between professional imaging and home monitoring. In this context, technical acquisition, acquisition adequacy, interpretive reliability, and downstream clinical decision-making should be understood as related but distinct steps. A patient may successfully complete image acquisition from a procedural standpoint while still producing images that are insufficient for reliable clinical interpretation. Conversely, even technically adequate images must remain sufficiently consistent and reproducible to support safe downstream clinical decisions.

Several emerging initiatives are exploring patient-operated ultrasound acquisition in structured clinical settings, particularly in fertility and obstetric monitoring. These include patient-operated transvaginal ultrasound systems for IVF monitoring, such as IMMA, as well as home ultrasound platforms designed for remote obstetric assessment, such as Pulsenmore. Similar concepts are also being explored in other areas including cardiac ultrasound monitoring and breast imaging, suggesting that redistribution of image acquisition may extend across multiple clinical specialties. The present analysis does not evaluate any specific technology but examines the broader regulatory and reimbursement implications of decentralized image acquisition models.

Fertility monitoring provides a particularly structured example in which this model can be examined (Box 1). In fertility medicine, measurement variability and interobserver differences in follicular assessment are already recognised challenges even within conventional clinician-operated imaging, illustrating that acquisition quality directly affects interpretability (14–16).

Box 1 **IVF monitoring as a structured first use case for patient-operated imaging.**

**Clinical context**

Ovarian stimulation monitoring in assisted reproductive technology (ART) is protocol-driven and time-bound. Ultrasound is repeatedly used to assess follicle number and size as well as endometrial thickness, guiding medication adjustments and trigger timing (16). Clinical decisions follow established algorithms based on serial measurements, while interpretation remains clinician-led.

**Why this pathway is structurally suitable**

Unlike exploratory diagnostic imaging, ART monitoring operates within a constrained framework:

- defined anatomy
- predictable timing windows
- repeated measurements over a short interval
- clear decision thresholds

In this setting, acquisition adequacy can be operationalized. Images either meet criteria for measurement or require repetition. The clinical task is structured monitoring rather than open diagnostic exploration.

**Regulatory implications**

Because anatomy and decision pathways are predefined, intended purpose can be narrowly framed around monitoring within fertility protocols. Risk analysis can therefore focus on acquisition adequacy and interpretive reliability rather than broad diagnostic claims.

**Health system implications**

Fertility monitoring typically requires multiple clinic visits over a short period. Decentralized acquisition could reduce visit burden without shifting clinical responsibility. However, reimbursement integration depends on reliable acquisition quality and avoidance of downstream inefficiencies such as repeat examinations with clinic catch-up visits or delayed clinical decisions.

**Policy relevance**

IVF monitoring therefore represents a bounded environment in which regulators and health systems can evaluate patient-operated acquisition before broader imaging applications are considered.

Regulatory and reimbursement implications of POICA remain largely unexplored. For regulators and payers, the central question is not whether patients can technically acquire images, but whether clinical interpretation can remain reliable when acquisition is decentralized.

While the structural questions raised by patient-operated imaging are relevant across health systems, this analysis focuses on Europe because the MDR provides a unified regulatory framework and European reimbursement models make the organization of the imaging act particularly explicit.

Examining these systems through the framework of the MDR and European reimbursement architecture helps clarify the conditions under which such models could realistically integrate into clinical practice.

## 3 Regulatory framework: MDR classification and acquisition adequacy

The MDR classifies devices based on intended purpose, invasiveness, duration, and the consequences of erroneous output. This structure works well when trained professionals operate within institutional safeguards.

As discussed above, the principal regulatory risk in POICA concerns **acquisition adequacy**: the clinician's ability to rely on the image for a decision. Three points follow:

1. **Intended purpose language matters.** Small shifts in wording can change classification expectations and evidence needs (17).
2. **Risk management must assume lay-user variability** as the default condition rather than an exceptional scenario. “Foreseeable use error” is not a corner case in home-use acquisition.
3. **Acquisition guidance becomes a safety control.** If software prompts, quality checks, or lockouts are used, they become part of the device's safety/performance claims (13, 18).

A partial regulatory analogy is self-testing *in vitro* diagnostics (IVDs) (*e.g.,* home INR, HIV self-testing), where lay users perform the technical act of sample acquisition and testing within a regulated framework designed to manage user variability and ensure interpretive reliability when required.

## 4 Usability and clinical reliability

Under MDR Annex I, usability is a safety requirement when use errors may cause harm (3). In devices intended for non-professional users, usability becomes a determinant of safety because user interaction itself becomes a potential source of error (19). Accordingly, MDR guidance emphasizes that devices intended for lay users must demonstrate safe and effective operation under foreseeable real-world conditions through appropriate usability validation (20).

Usability engineering and risk management standards set the structure (1, 2, 20). In POICA, usability determines whether lay users can reliably produce images that meet predefined adequacy criteria under real-world conditions. Successful procedural completion alone is insufficient if image quality does not support reliable interpretation and downstream clinical decision-making.

Software-guided acquisition can reduce variability, but it creates a second dependency: prompts and quality alerts must be understood and acted upon. FDA human factors guidance emphasizes that “successful use” must be demonstrated through realistic user testing (21).

In IVF monitoring, this is concrete. If image quality drifts, clinical decisions drift. Usability evidence is the bridge between regulatory compliance and clinician trust.

## 5 Liability and responsibility in decentralized imaging

Liability concerns drive adoption even when they are not written into reimbursement rules. In conventional imaging, acquisition and interpretation occur inside the same institutional framework, and responsibility lines are familiar. POICA changes that configuration without transferring clinical authority.

- **Manufacturers** remain responsible for foreseeable misuse in home-use contexts; clear adequacy thresholds and safeguards reduce ambiguity.
- **Clinicians** retain interpretive accountability.
- **Patients** perform technical acquisition steps without assuming diagnostic responsibility.

The liability issue arises when technical completion does not translate into a clinically usable acquisition. If adequacy thresholds are explicit and auditable, liability becomes manageable. If not, adoption slows regardless of CE marking.

**Figure 2:**
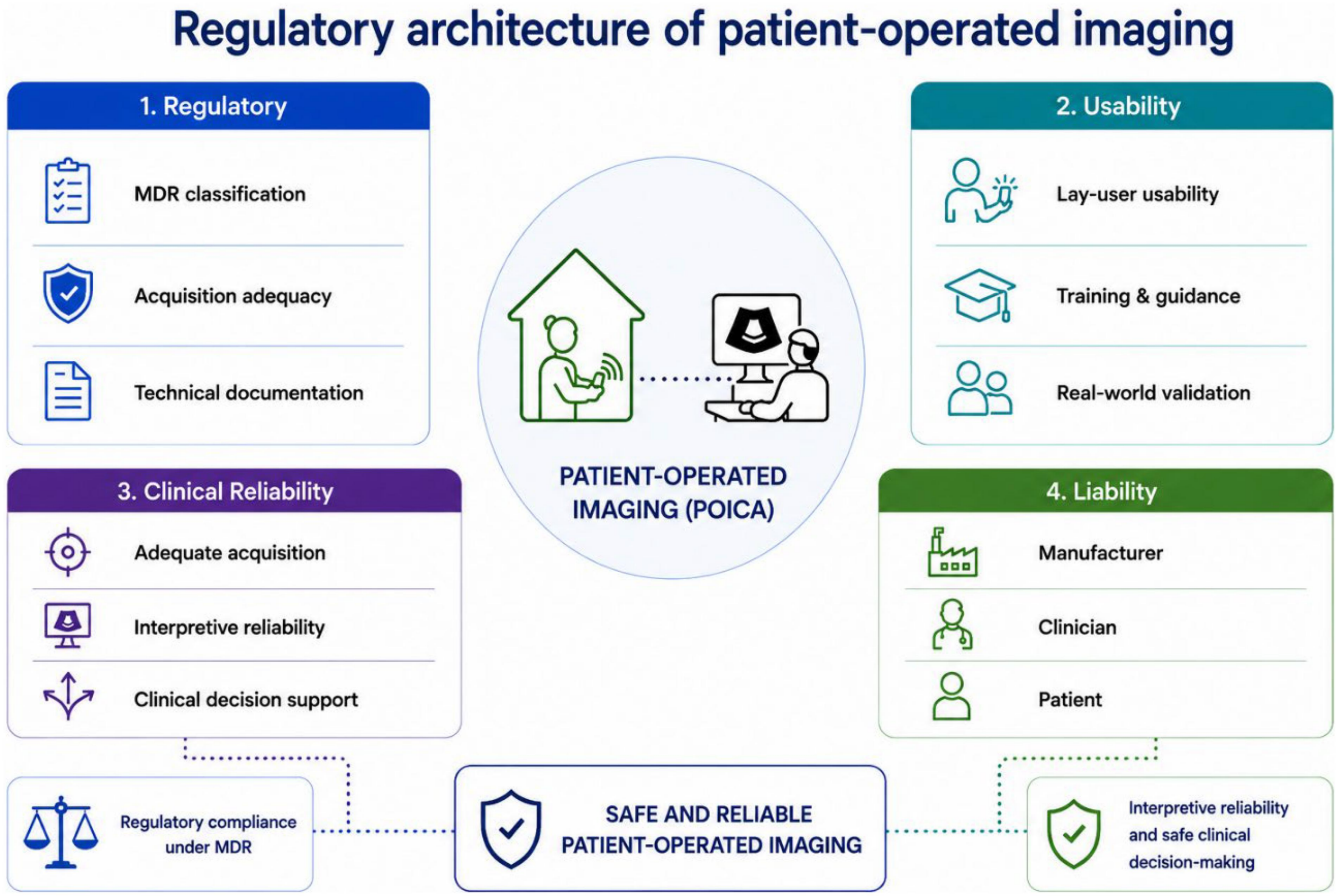
Regulatory architecture of patient-operated imaging. When image acquisition is performed by patients, regulatory evaluation extends beyond technical device performance alone. The framework illustrated here highlights four interconnected domains relevant to decentralized acquisition models: regulatory classification and acquisition adequacy under MDR requirements; usability validation and human-factors engineering for lay users; preservation of clinical reliability, including interpretive reliability and downstream clinical decision support; and allocation of responsibilities among manufacturers, clinicians, and patients. Together, these elements determine whether patient-operated imaging can maintain safe and reliable clinical interpretation within decentralized care pathways.

These three elements illustrate how regulatory risk emerges from changes in imaging workflow (Figure 2).

Variability in medico-legal frameworks across European countries further illustrates the complexity of responsibility allocation in imaging. In many jurisdictions, physicians are traditionally bound by an obligation of means, requiring them to provide care consistent with accepted professional standards rather than to guarantee a specific outcome. In radiology, however, expectations regarding diagnostic accuracy are high, and the interpretation of responsibility may vary depending on national legal and professional frameworks (22). This variability highlights that responsibility in imaging is not solely determined by who performs acquisition, but by whether the overall process meets standards of reliability and clinical adequacy.

In addition, the physical separation of image acquisition and interpretation is already established in routine practice through teleradiology. Radiologists frequently interpret studies acquired at distant sites, relying on images obtained by other operators without direct control over acquisition conditions. Even within institutional settings, acquisition and interpretation are often performed by different professionals. Patient-operated imaging therefore does not introduce a fundamentally new separation but extends an existing model in which interpretive responsibility is maintained despite variability in acquisition. The key question remains whether acquisition conditions, regardless of the operator, allow for reliable clinical interpretation.

## 6 Assessment of policy and reimbursement implications

In European health systems, regulatory approval and reimbursement follow different logics. CE marking confirms safety and performance under an intended use but does not confer coverage. European HTA frameworks typically evaluate technologies across clinical, organizational, and economic domains, as reflected in the EUnetHTA Core Model (23).

Different HTA organizations may approach patient-operated imaging through distinct evaluative frameworks. As highlighted in recent analyses of HTA methodologies for diagnostic technologies, assessment approaches and evidence requirements vary across systems depending on organizational structure, reimbursement integration, and the role of HTA within national healthcare decision-making processes (24). Because POICA modifies both the technical execution and organization of imaging care, evaluation is likely to extend beyond diagnostic performance alone to include operational and implementation-related considerations.

In practice, HTA evaluations of patient-operated imaging may require broader assessment domains than those traditionally applied to imaging devices performed entirely within institutional settings. Relevant evaluation domains may include:

- Acquisition reliability:
  ○ clinically adequate acquisition rates, repeat examination rates, and robustness under real-world conditions;
- Clinical workflow integration:
  ○ implementation feasibility, clinician review burden, preservation of physician oversight, and impact on downstream clinical decision-making;
- Patient-related factors:
  ○ patient training requirements, usability under home conditions, and patient-reported experience;
- Health-system impact:
  ○ number of avoided clinic visits, workflow redistribution, and effects on healthcare resource utilization.

These dimensions are likely to influence coverage decisions because the organization of the imaging act itself becomes part of the value assessment.

Coverage decisions ultimately require demonstration of both clinical benefit and economic value (25). **Economic evaluation** examines whether a decentralized acquisition model generates sufficient value relative to current practice. A formal economic evaluation would require empirical data on how decentralized acquisition modifies the care pathway in practice, for example through cost-effectiveness analysis comparing the new model with current care. In structured settings such as IVF stimulation monitoring, this could be assessed by comparing conventional clinic-based monitoring with patient-operated acquisition in terms of clinical reliability of the resulting images, number of in-person visits, clinician time devoted to monitoring, and overall resource use across the cycle. Such data would allow comparison of the cost and operational implications of the two monitoring approaches under real clinical conditions. Evidence of this type would be necessary for HTA bodies and payers to determine whether decentralized acquisition generates sufficient value relative to existing monitoring models.

Once economic value has been demonstrated, **reimbursement architecture** will determine how providers are compensated and incentivized. The present analysis focuses primarily on the structural implications for reimbursement integration rather than on formal economic evaluation of the model itself.

Imaging reimbursement is traditionally organized around a unified encounter in which acquisition and interpretation are usually performed and reimbursed together. Health systems have already adapted to partial separation of acquisition and interpretation through the development of teleradiology. In this model, radiologists interpret images acquired at distant sites, often without direct involvement in the acquisition process. Reimbursement frameworks have evolved to recognize this organizational separation while maintaining physician responsibility for interpretation (11). However, in teleradiology, acquisition remains performed by trained healthcare professionals, and the structure of the imaging act is preserved. Patient-operated imaging extends this separation further by redistributing the technical act of acquisition to patients, thereby challenging reimbursement models more directly. The question for reimbursement systems is therefore not whether acquisition and interpretation can be separated, but how far this separation can extend while preserving clinical reliability and accountability.

Health systems have dealt with similar structural shifts when technical execution moved outside institutions, such as home dialysis or remote monitoring models (26, 27). In those domains, reimbursement evolved to recognize distributed workflows while preserving centralized medical oversight.

Ultimately, reimbursement decisions hinge on whether the model improves outcomes or operational efficiency sufficiently to justify coverage. Emerging systems built around a bounded fertility pathway make this reimbursement question concrete without implying broader diagnostic decentralization.

When reimbursement frameworks do not match a new care model, European systems typically introduce bounded adjustments such as pilot funding, add-on payments, pathway-specific codes, bundled care models, or remote monitoring reimbursement structures. The mechanisms used to integrate decentralized care models may nevertheless differ across European systems depending on reimbursement architecture and organizational frameworks. These differences may influence how responsibility and physician oversight are interpreted when acquisition occurs outside conventional institutional settings. Remote monitoring reimbursement debates in Europe show how payment can evolve to recognize distributed workflows when value and operational feasibility are demonstrated (26, 28).

A further dimension concerns physician acceptance. The proposed model modifies the traditional workflow in which clinicians perform both acquisition and interpretation. For some physicians, the acquisition stage is not only a technical step but also an integral component of clinical assessment and professional practice. Removing this component may therefore generate resistance independent of financial considerations. Adoption will depend on whether the new workflow preserves clinical oversight and maintains diagnostic confidence while integrating into existing clinical routines, as clinician acceptance of new technologies often depends on how they fit within established professional practices (29, 30). In practice, decentralized acquisition will only be accepted if physicians perceive that it supports rather than fragments their clinical role. In addition, the redistribution of acquisition raises practical questions about compensation. If physicians no longer perform the technical component of imaging, interpretive and supervisory roles must remain appropriately recognized within the care pathway.

Reimbursement mechanisms must then recognize the redistribution of tasks within the imaging workflow. In POICA, physicians retain responsibility for interpretation and clinical decision-making even though acquisition occurs outside the clinical encounter. Payment structures must therefore ensure that the interpretive and supervisory components of the medical act remain appropriately recognized. The objective is not to create an additional reimbursed act, but to adapt existing reimbursement frameworks to a model in which acquisition and interpretation no longer occur in the same clinical setting.

Although reimbursement systems differ across European countries, they share a common structural feature: payment is typically attached to clearly defined professional acts. POICA requires reimbursement systems to recognize decentralized acquisition while maintaining a single accountable clinical act.

This challenge is manageable if three conditions are met: reliable acquisition, a clear allocation of responsibilities, and evidence that the model reduces system burden rather than shifting it elsewhere. Reimbursement schemes must recognize that decentralized acquisition changes the distribution of clinical tasks and may require adjustments to ensure that physician incentives remain aligned with high-quality care.

## 7 Actionable recommendations

Based on this analysis, several conditions appear necessary for the integration of patient-operated imaging into European health systems:

- Acquisition adequacy criteria should be explicitly defined, validated, and monitored in real-world use;
- Responsibility boundaries must remain clearly specified when acquisition is performed outside clinical settings;
- HTA evaluations of patient-operated imaging should assess not only diagnostic performance, but also acquisition reliability, workflow integration, implementation feasibility, physician review burden and redistribution of clinical responsibilities;
- Reimbursement frameworks should recognize the separation of acquisition and interpretation without duplicating payment structures while preserving physician accountability for clinical interpretation;
- Clinical workflows should preserve physician oversight and diagnostic confidence.

These elements provide a practical basis for evaluating decentralized imaging models within existing regulatory and reimbursement systems.

## 8 Discussion

This article is a conceptual and policy-focused analysis rather than an evidence-based assessment of clinical safety, effectiveness, or cost-effectiveness. It is intended to provide a framework for examining how decentralized imaging acquisition models may interact with existing regulatory, organizational, and reimbursement systems, and should not be interpreted as evidence supporting clinical implementation or economic value.

POICA represents a structural reconfiguration of the clinical act rather than a marginal extension of home monitoring. MDR pathways can authorize such systems, but policy success depends on three concrete elements:

- **Adequacy thresholds** that are explicit, validated, and monitored in real use
- **Responsibility boundaries** that remain stable when acquisition moves outside institutions
- **System-level performance** that preserves interpretive quality without creating inefficiency

Decentralized acquisition may also introduce risks related to inadequate image acquisition, delayed recognition of clinically significant findings, increased need for repeat examinations, patient training variability, and potential workflow fragmentation. These risks require empirical evaluation and should be considered alongside potential organizational and accessibility benefits.

In this context, evidence generated from defined clinical pathways will be particularly important to determine whether decentralized acquisition can maintain reliability in routine care. IVF monitoring represents a sensible first use case because it is bounded and protocol driven. It allows regulators and payers to test whether decentralized acquisition can be dependable before broader imaging applications are considered.

### Key message

The policy challenge is not technological feasibility but institutional adaptation to a model in which image acquisition and clinical interpretation are no longer co-located.
